# Salivary and Lacrimal Gland Alterations of the Epidermal Fatty Acid-Binding Protein (E-FABP) in Non-Obese Diabetic Mice †

**DOI:** 10.3390/ijms23073491

**Published:** 2022-03-23

**Authors:** Murat Dogru, Takashi Kojima, Cem Simsek, Taeko Nagata, Kazuo Tsubota

**Affiliations:** 1 Department of Ophthalmology, Keio University School of Medicine, Tokyo 160-8582, Japan; kojkoj@me.com (T.K.); cemsimsekmd@gmail.com (C.S.); tngtax4@gmail.com (T.N.); tsubota@tsubota-lab.com (K.T.); 2 Department of Ophthalmology, Mugla Sitki Kocman University School of Medicine, Mugla 48000, Turkey; 3 Tsubota Laboratory, Inc., Tokyo 160-0016, Japan

**Keywords:** Sjögren syndrome, NOD mouse, dry eye, lacrimal gland, salivary gland

## Abstract

The purpose of this study was to investigate the changes in E-FABP in the salivary and lacrimal glands of the Sjögren syndrome (SS) model non-obese diabetic mice (NOD). Cotton thread and ocular vital staining tests were performed on 10-week NOD male mice (*n* = 24) and age- and sex-matched wild-type (WT) mice (*n* = 25). Tear and saliva samples were collected at sacrifice for E-FABP ELISA assays. Salivary and lacrimal gland specimens underwent immunohistochemistry stainings for E-FABP. Real-time RT-PCR was also performed for the quantification of mRNA expression levels in the salivary and lacrimal glands. Corneal vital staining scores in the NOD mice were significantly higher compared with those for the wild-type mice (*p* = 0.0001). The mean tear E-FABP level showed a significantly lower concentration in the NOD mice (*p* = 0.001). The mean saliva E-FABP level also showed a significantly lower concentration in the NOD mice (*p* = 0.04). Immunohistochemistry revealed intense E-FABP staining in the LG acinar epithelium and less intense staining in the acinar epitheliae of the SGs in the NOD mice compared to the WT mice. Real-time RT-PCR for the mRNA expression of E-FABP showed a significantly decreased expression in the SG and a significant increase in the LG of the NOD mice compared to the WT mice. In conclusion, the E-FABP showed marked alterations in the tear film, saliva, lacrimal, and salivary glands of the NOD mouse, which may help explain the ocular surface changes in relation to the dry eye disease in this SS model mouse and keratoconjunctivitis sicca in SS patients.

## 1. Introduction

Sjögren syndrome (SjS, SS) is a long-term autoimmune disease that affects the body’s moisture-producing (lacrimal and salivary) glands and often seriously affects other organ systems, such as the lungs, kidneys, and nervous system. While the exact cause is unclear, it is believed to involve a combination of genetics and an environmental trigger, or to occur independently of other health problems (primary Sjögren’s syndrome) or as a result of another connective tissue disorder (secondary Sjögren’s syndrome) such as rheumatoid arthritis (RA), systemic lupus erythematosus (SLE), or systemic sclerosis. The inflammation that results, mainly from lymphocytic infiltrates, progressively damages the glandular structures. While Sjögren’s syndrome is one of the most common autoimmune diseases, it has no specific and non-invasive diagnostic tests. Diagnosis is made by biopsy of the moisture-producing glands and blood tests for specific antibodies [1,2,3]. Research on the relationship between SS and FABP includes a study by Baldini et al. which found, through the use of proteome analysis of human saliva, that there was a highly significant difference in the FABP5 expression in the SS patient group compared with the healthy volunteer group. They also mentioned the possibility that FABP5 could be used as a specific marker that reflects the disease activity of SS and its severity [4].

The fatty-acid binding family of proteins (FABP) have been reported as useful and novel markers in the diagnosis of oxidative stress–related disorders, including acute renal failure, myocardial infarction, and Alzheimer’s disease [5,6,7]. At least nine molecular species of FABP have been identified, all having different expressions in different organs [8]. The FABP molecules act as intracellular chaperones with the ability to bind to insoluble long-chain unsaturated fatty acids such as arachidonic acid and DHA. FABPs are involved in a variety of intracellular functions by making these fatty acids soluble [9,10,11]. Among them, E-FABP has been shown to be related to the immune system, oxidative stress, tissue inflammation, cell differentiation and programmed death, and malignant diseases [12,13,14,15,16,17,18].

Our previous research suggested that the accumulation of lipid peroxidation products caused damage to the lacrimal glands (LG) in patients with SS and that oxidative stress played a role in the pathogenesis of dry eye disease [19]. Since tissue samples from exocrine glands in human SS subjects are indeed difficult to acquire due to ethical reasons, in order to study the alterations of these proteins and their relation to the pathogenesis of dry eye disease, we chose to use the NOD mouse (a model mouse of SS) to investigate the changes in E-FABP (FABP5), which is expressed in the oral mucosa, ocular surface, and exocrine glands, in an attempt to understand its role in the pathogenesis of dry eye disease. The tear film, salivary and lacrimal gland changes in the NOD mice were also compared with those in the WT mice. 

## 2. Results

### 2.1. Aqueous Tear Production Alterations in the NOD and Wild Type Mice

**Figure 1:**
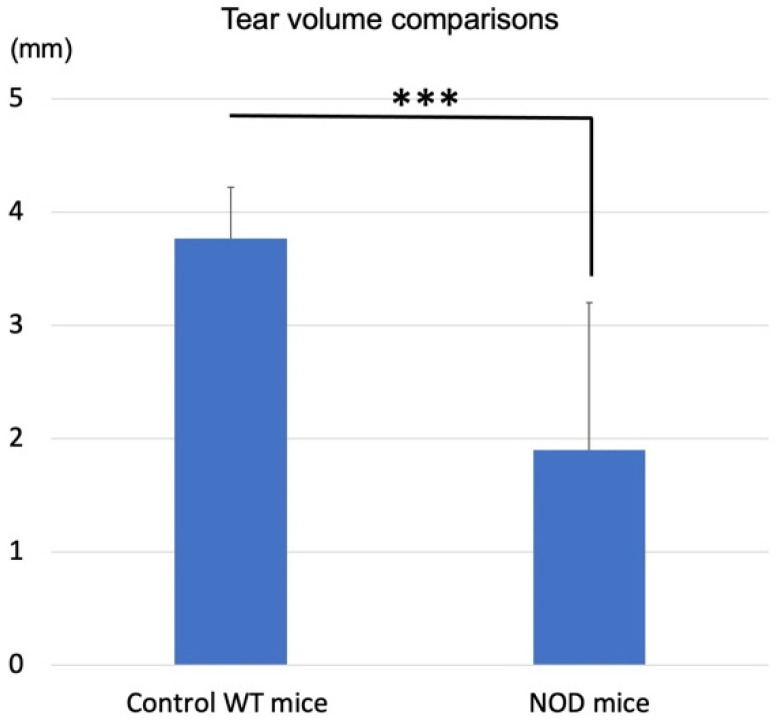
Comparison of tear volume between wild-type (WT) and non-obese diabetic (NOD) mice. Tear volume in the NOD mice was significantly lower than in the WT mice. *** represents *p* < 0.0001.

The mean aqueous tear production was significantly lower in the *NOD* mice compared to the WT mice (*p* < 0.0001) (Figure 1).

### 2.2. Corneal Vital Staining and Tear Film Stability Changes

**Figure 2:**
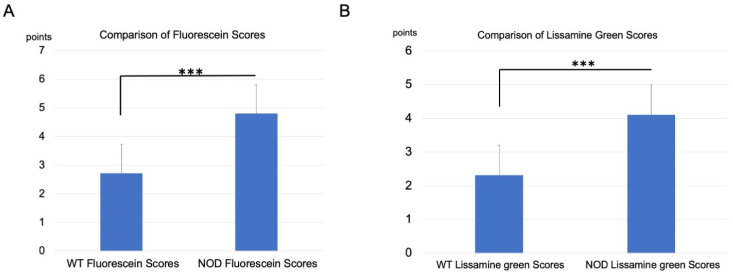
Comparison of vital staining scores between the wild-type (WT) and NOD mice. (**A**) Fluorescein staining scores in the NOD mice were significantly higher than those in the WT mice (*p* < 0.0001). (**B**) Lissamine green staining scores in the NOD mice were also significantly higher than those in the WT mice (*p* < 0.0001). *** represents *p* < 0.0001.

The mean corneal vital staining scores were significantly worse in the *NOD* mice compared to the WT mice (*p* < 0.0001) (Figure 2).

### 2.3. Comparison of E-FABP Concentration in the Tears and Saliva

**Figure 3:**
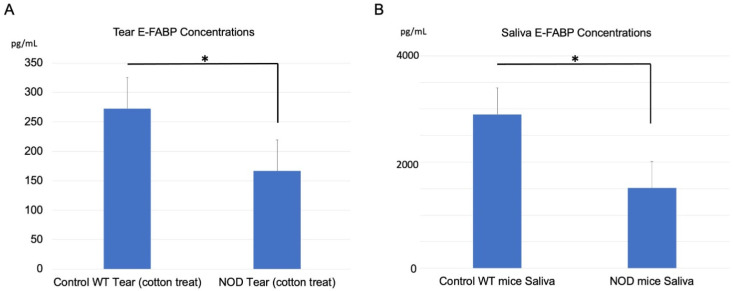
Comparison of E-FABP concentrations in tears and saliva between the wild-type (WT) mice and the NOD mice. (**A**) Tear E-FABP concentration in the NOD mice was significantly lower than in the WT mice. (*p* = 0.0135). (**B**) Saliva E-FABP concentration in the NOD mice was also significantly lower than in the WT mice. (*p* = 0.04). * represents *p* < 0.05. E-FABP: epidermal fatty acid binding protein.

The mean E-FABP concentration in the tears was 275 ± 50 pg/mL in the WT mice and 175 ± 50 pg/mL in the NOD mice; the difference between the groups was statistically significant (*p* = 0.0135) (Figure 3A). The E-FABP concentration in the saliva was 2800 ± 600 pg/mL in the WT mice and 1500 ± 500 pg/mL in the NOD mice. This difference was also statistically significant (*p* = 0.04, Figure 3B).

### 2.4. Comparison of E-FABP Marker Immunohistochemistry Stainings

**Figure 4:**
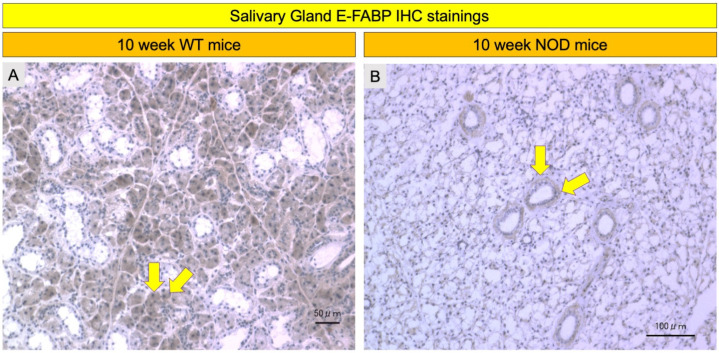
Comparison of E-FABP immunohistochemistry in the salivary gland between the wild-type (WT) mice and the NOD mice. Representative immunohistochemistry images showed less intense immunostaining in the acinar epithelium of the NOD mice (**B**) when compared with the WT mice (**A**) (yellow arrows). IHC: immunohistochemistry.

**Figure 5:**
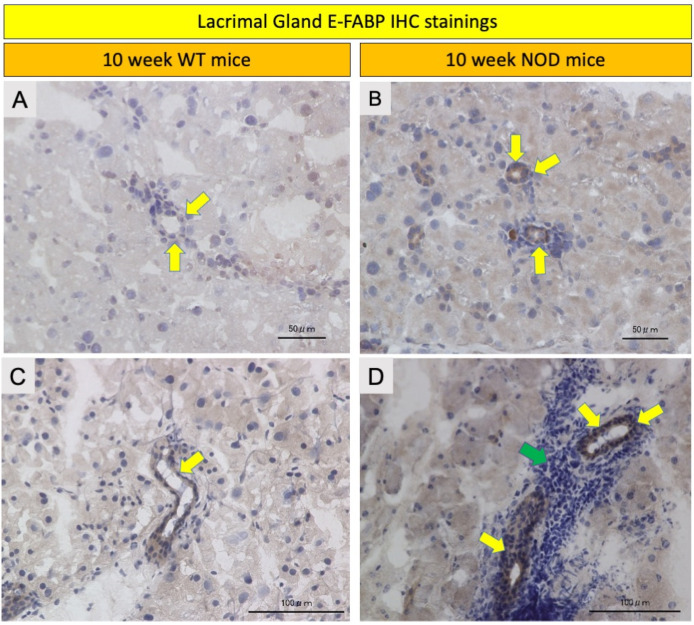
Comparison of E-FABP immunohistochemistry in the lacrimal glands of the wild-type (WT) mice and the NOD mice. Representative immunohistochemistry images showed increased immunostaining of the acinar epithelium in the NOD mice (**B**,**D**) compared to the WT mice (**A**,**C**) (yellow arrows). Moreover, a denser infiltration of inflammatory cells around the acinar units of the NOD mice compared to the WT mice can be observed (green arrow). IHC: immunohistochemistry.

**Figure 6:**
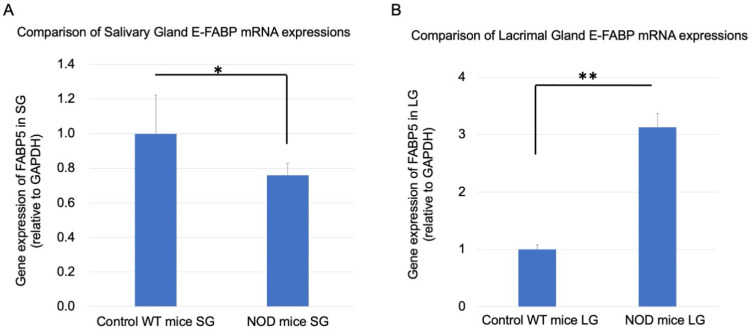
Comparison of E-FABP mRNA expressions in the salivary and lacrimal glands of the wild-type (WT) and the NOD mice. (**A**) In the salivary glands, the E-FABP mRNA expression in the NOD mice was significantly lower than that in the WT mice (*p* = 0.001). (**B**) In the lacrimal glands, the E-FABP mRNA expression in the NOD mice was significantly higher than that in the WT mice (*p* = 0.0008). * and ** represent *p* < 0.05 and *p* < 0.001, respectively. SG: salivary gland, LG: lacrimal gland, GAPDH: glyceraldehyde phosphate dehydrogenase.

E-FABP immunoreactivity was observed in the acinar epitheliae of the salivary and lacrimal glands (LG) in both the WT and *NOD* mice (Figure 4 and Figure 5). The salivary gland acinar epithelium showed markedly less staining in the NOD mice compared to the WT mice (Figure 6A,B). The acinar epithelium of the LGs in the NOD mice showed intense staining with E-FABP antibodies compared to the WT mice (Figure 4A,B). The LGs in the NOD mice also showed denser inflammatory cell infiltrates around the secretory acini compared to the WT mice (Figure 5C,D).

### 2.5. Comparison of Real-Time RT-PCR Lacrimal and Salivary Gland E-FABP mRNA Expressions

Real-time RT-PCR for the mRNA expression of E-FABP showed a significantly decreased expression in the SGs of the NOD mice (*p* = 0.001) compared to the WT mice (Figure 6A) and a three-fold significant increase in the LG of the NOD mice compared to the WT mice (Figure 6B) (*p* = 0.0008).

## 3. Discussion

Employing the proteome analysis of human saliva, Baldini et al. initially reported a highly significant difference in the FABP5 expression in primary SS patients compared with healthy volunteers, raising the possibility that FABP5 might have uses as a specific marker that reflects the disease activity and/or severity [4]. In a previous study by us, the E-FABP concentration in tears of primary SS patients was found to be significantly lower than in healthy control subjects. The E-FABP concentration in tears was also observed to significantly correlate with decreased tear quantity and tear film break-up time, as well as with increased conjunctival and corneal vital staining scores. This study suggested that the E-FABP concentration in tears was related to ocular surface epithelial damage and tear instability and might be a promising novel biomarker in the diagnosis of SS [20]. Since ethical issues strongly hamper the collection and study of lacrimal and other exocrine gland tissues in humans in order to investigate the pathogenetic mechanisms, we had not been able to look into the lacrimal and salivary gland changes in this protein before. Animal models of SS or dry eye disease help us to analyze many pathogenetic relations from which fruitful conclusions can be drawn for similar human diseases [21].

Lacrimal gland infiltration has been reported to develop earlier in SS patients relative to salivary gland inflammation [22], which suggests the possibility that ocular surface disease may represent the early or initial phases of SS. Animal model studies including the ocular spectrum of SS can be very useful for developing early interventions in SS and can facilitate our understanding of how LG pathology in SS affects the ocular surface tissues [23,24]. In this context, Gonzales et al. suggested an ocular vital staining score as a diagnostic test with a high specificity and sensitivity to differentiate SS–related ocular surface disease [25]. Increased ocular surface vital staining scores reflect the perturbation of the kerato-conjunctival epithelial barrier. An investigation of ocular surface disease in mouse models of SS in conjunction with LG pathology may provide insight into the disease progression or pathogenesis of the underlying disorders. NOD mice have been reported to have similar ocular surface disease and dry eye features to human ocular surface disease, including decreased tear volume, the loss of corneal barrier integrity (increased staining scores), reduced tear mucins, and increased lacrimal gland inflammatory infiltrates [21], which led us to choose NOD mice to examine FABP changes in the tears, saliva, lachrymal and salivary glands in this study. Similar to previous reports, we found significantly decreased tear secretion and significantly increased corneal vital staining scores in the NOD mice in comparison to age- and sex-matched WT mice. A PubMed search using the key words “FABP”, “tear”, and “saliva” revealed that tear and saliva concentrations of E-FABP had not been studied in NOD mice before. This study found a significant decrease in both the tear and saliva E-FABP concentrations in the NOD mice when compared with the WT mice. The salivary gland E-FABP mRNA expression was significantly reduced in the NOD mice. These findings were also backed up by IHC stainings showing less intense acinar epithelial staining in the salivary glands of NOD mice compared with the WT mice. These findings were in alignment with Baldini’s proteomic analysis findings of decreased saliva E-FABP in SS patients. As expected, we found a more intense inflammatory cell infiltration around the acini and ducts of LGs in the NOD mice in comparison to the WT mice. To our surprise, we found an increase in the E-FABP mRNA expression in the NOD LGs when compared with the LGs of the WT mice.

E-FABP has been reported to perform regulatory roles in the immune system, oxidative stress responses, the inflammatory response of tissues, cell differentiation and apoptosis, and malignant diseases [12,13,14,15,16,17,18,26]. E-FABP was initially found in rat skin epithelial cells [12], which allowed the rapid advancement of research on E-FABP in dermatology literature. Studying the FABP5 knockout mice, Owada et al. identified an important function of FABP5 in the moisture barrier of skin [26]. Possible sources of E-FABP in tears include the ocular surface epithelium, meibomian, and sebaceous and/or lachrymal glands. E-FABP on the ocular surface tear film–lachrymal gland unit might play a similar role as the skin, which may be the maintenance of the ocular surface epithelial barrier and trans-epithelial water transport. A decrease in E-FABP might lead to disturbances in the ocular surface epithelial integrity (as evidenced by the significantly higher extent of corneal epithelial damage in the NOD mice), which may lead to increased tear evaporation and dry eyes. A previous investigation conducted by us in primary SS patients revealed an increased lipid peroxidation with an elevation of early and late lipid oxidative stress markers such as HEL and 4-HNE in the conjunctival tissue [19]. Since E-FABP is known to play a role in the regulation of oxidative stress, we hypothesize that the decreased tear concentration of this protein may be due to an exaggerated transport of the free fatty acids and cholesterol into the ocular surface in an attempt to balance the oxidative stress and repair the damage in the epithelial cell membranes. This hypothesis needs to be backed up by a determination of corneal tissue expressions of lipid peroxidation markers as well as corneal free fatty acid and cholesterol-level analyses in the NOD mice in future studies. The increased E-FABP mRNA expression in the lachrymal glands may be an early response in the LGs to compensate for the decline of this essential “ocular surface epithelial homeostasis helper” protein in the tear film or an associated lachrymal gland finding in conjunction with increased peri-acinar and periductal inflammatory cell infiltration. It is also possible that the LGs may be producing more FABP (as evidenced by the intense immunostaining and increased FABP mRNA expression), but the secretory mechanism could have been shut down or be defective, resulting in FABP not being released into the tears. The secretory pathways of FABP from LGs in the NOD mice compared to WT mice also need further investigation.

An investigation into the time-wise alterations of E-FABP beyond 10 weeks during the natural course and development of ocular surface disease till senescence in the NOD mice will add invaluable information to the existing literature. In addition, further research into the identification and quantification of the resident memory type of T cells among other inflammatory cells in the LGs of NOD mice would be interesting since these T cells utilize exogenous free fatty acids in the beta-oxidation within mitochondria, which in turn would stimulate an increase in the FABP5 uptake of free fatty acids to be transferred to an ailing ocular surface epithelium [27].

Furthermore, analyses of time-wise changes in the LG and SG PPAR gamma levels and the activation markers of the NF-κB pathway may pave the way to an enhanced understanding of the pathogenesis of E-FABP involvement in the SS-related dry eye disease since E-FABP ligands may act as peroxisome proliferator-activating receptor γ (PPARγ) ligands during states of increased inflammation [28], and PPARγ is known to induce the expression of the gene associated with cholesterol transportation [29] and to regulate the transcription activation of pro-inflammatory genes in macrophages.

## 4. Materials and Methods

### 4.1. Animals

Twenty-four ten-week-old *NOD* male mice with an ICR-JCL background and twenty five age- and sex-matched Balb/c strain wild-type (WT) mice were examined. *NOD* and WT mice were purchased from Japan Clea (Osaka, Japan). All studies were performed in accordance with the Association for Research in Vision and Ophthalmology (ARVO) Statement for the Use of Animals in Ophthalmic and Vision Research.

### 4.2. Aqueous Tear Production Measurements

Phenol red-impregnated cotton threads (Zone-Quick, Showa Yakuhin Kako Co., Ltd., Tokyo, Japan) were used to measure aqueous tear production without anesthesia. Briefly, cotton threads were immersed into the tear meniscus in the lateral canthus of the mice eyes for 60 s and the length of the thread wetted was measured in millimeters. The tear production was weight-adjusted by dividing the amount of total aqueous tears produced in 60 s by their weight.

### 4.3. Collection of Tears, Saliva, and Serum

Tears and saliva were collected from all mice during the initial examination. Ten microliters of tears were collected from the lateral canthus using glass microcapillary tubes. Saliva was individually collected by aspiration from all the mice directly from their mouths with a micropipette. Saliva samples were frozen immediately in liquid nitrogen and stored at −80 °C until required. Before protein quantification, the samples were centrifuged at 16,000× *g* for 5 min at 4 °C to remove particulate matter and salivary proteins that could be precipitated. Only the soluble fraction was used for further analyses [30]. The tear samples underwent immediate centrifugation at 3000 rpm and 4 °C for 5 min with the supernatants stored at −80 °C until analysis. The E-FABP concentration in the resulting supernatants was measured using the FABP5 ELISA Kit (Human) (Aviva Systems Biology, San Diego, CA, USA) based on the standard sandwich ELISA technique and following the manufacturer’s instructions.

### 4.4. Enzyme-Linked Immunosorbent Assay for E-FABP

All specimens were thawed, subjected to vortex, and centrifugally separated, and finally, all precipitates were removed prior to assaying. The assays were performed using the FABP5 ELISA Kit (mouse) (LS Bio, Seattle, WA, USA) based on the standard sandwich ELISA technique and following the manufacturer’s instructions.

### 4.5. Corneal Epithelial Cell Damage Evaluation

A total of 2 μL of 0.5% sodium fluorescein and 1% LG was instilled into the conjunctival sac. Corneal epithelial damage was assessed after 2 min of fluorescein dye instillation. Fluorescein staining tests were conducted using a hand-held slit lamp biomicroscope using cobalt blue light (Kowa, Tokyo, Japan). In fluorescein and Lissamine green stainings, the mice corneas were divided into 3 equal upper, middle, and lower zones. Each zone had a staining score ranging between 0 and 3 points, with the minimum and maximum total staining scores ranging between 0 and 9 points. The presence of scarce staining in 1 zone was scored as 1 point, whereas punctate staining covering the entire zone was scored as 3 points, as previously described.

### 4.6. Immunohistochemistry Staining for E-FABP

The peroxidase system Vectastain ABC kit (rabbit IgG; Vector Laboratories, Burlingame, CA, USA) and the anti-FABP5 (D1A7T) rabbit monoclonal antibody (Cell Signaling Technology, Danvers, MA, USA) diluted with goat blocking serum at 1:200 were used. Tissue sections were fixed with 4% Paraformaldehyde for 5 min at room temperature, then incubated with normal goat serum (Vector Laboratories, Burlingame, CA, USA) for 2 h and 20 min at room temperature to block nonspecific background staining. The tissues were then treated with the anti-FABP5 rabbit monoclonal antibody for 2 h at room temperature. For the negative controls, the primary antibody was replaced with the rabbit IgG isotype control at the same concentration of the primary antibody (ab37415) (ABCAM, Cambridge, UK). Sections were then blocked using 0.03% H_2_O_2_ in methanol for 30 min. The tissue samples were treated with the biotin-labeled goat anti-rabbit IgG (Vector Laboratories) for 30 min, followed by an avidin-biotin-peroxidase complex treatment (Vector Laboratories, Burlingame, CA, USA) for 30 min. The sections were then washed in PBS, developed in a prepared DAB chromogen solution (Vector Laboratories, Burlingame, CA, USA), lightly counterstained with hematoxylin for 3 min at room temperature, washed with tap water, dehydrated, and mounted. Sections were then evaluated and imaged using an Axioplan2 imaging microscope (Carl Zeiss, Jena, Germany).

### 4.7. Real-Time PCR for Lacrimal and Salivary Gland E-FABP mRNA Expression

The total RNA was isolated from the lacrimal gland and salivary gland using an RNA extraction reagent (ISOGEN; Nippon Gene, Tokyo, Japan), according to the manufacturer’s instructions. The RNA was used for reverse transcription, and then cDNA synthesis was performed using the ReverTra Ace qPCR RT kit (TOYOBO, Osaka, Japan). A SYBR Green-based quantitative real-time PCR was performed using the Step One Plus System (Applied Biosystems, Framingham, MA, USA). The expression levels of mRNA were evaluated using the ΔΔCt method and normalized to the level of glyceraldehyde-3-phosphate dehydrogenase (GAPDH). Each PCR amplification was performed using a specific primer set. The primer sequences were as follows: GAPDH, 5′-TGTGTCCGTCGTGGATCTGA-3′ (sense) and 5′-CCTGCTTCACCACCTTCTTGAT-3′ (antisense); E-FABP, 5′-GACGACTGTGTTCTCTTGTAACC-3′ (sense) and 5′-TGTTATCGTGCTCTCCTTCCCG-3′ (antisense).

### 4.8. Statistical Analysis

A *t*-test was used for the statistical analyses employing the GraphPad Prism Software (San Diego, CA, USA). A *p* value of less than 5% was regarded as statistically significant.

## 5. Conclusions

This study provided distinct and descriptive alterations of E-FABP in the tear film and salivary and lachrymal glands of NOD mice, which might provide an explanation for ocular surface disease in SS. The concomitant identification of LG and SG E-FABP changes together with the quantification of inflammatory cell subtypes, free fatty acid, and cholesterol level changes in the ocular surface and the tear film and the investigation of the PPAR gamma and NF-κB pathway activation status remain future goals of this ongoing research project. Such studies may provide further evidence for the possibility of E-FABP being used as an early disease marker or provide evidence regarding the possibility of E-FABP being a severity or activity marker in SS.

## Data Availability

Not applicable.
